# Evaluation of brain stem auditory evoked potentials in stable patients with chronic obstructive pulmonary disease

**DOI:** 10.4103/1817-1737.42271

**Published:** 2008

**Authors:** Prem Parkash Gupta, Sushma Sood, Atulya Atreja, Dipti Agarwal

**Affiliations:** *Department of Respiratory Medicine, Postgraduate Institute of Medical Sciences, Rohtak, India*; 1 *Department of Physiology, Postgraduate Institute of Medical Sciences, Rohtak, India*

**Keywords:** Brainstem auditory evoked potentials, chronic obstructive pulmonary disease, correlation analysis, Mini–Mental Status Examination

## Abstract

Though there are few studies addressing brainstem auditory evoked potentials (BAEP) in patients with chronic obstructive pulmonary disease (COPD), *subclinical* BAEP abnormalities in stable COPD patients have not been studied. The present study aimed to evaluate the BAEP abnormalities in this study group.

**MATERIALS AND METHODS:**

In the present study, 80 male subjects were included: COPD group comprised 40 smokers with stable COPD with no clinical neuropathy; 40 age-matched healthy volunteers served as the control group. Latencies of BAEP waves I, II, III, IV, and V, together with interpeak latencies (IPLs) of I-III, I-V, and III-V, and amplitudes of waves I-Ia and V-Va were studied in both the groups to compare the BAEP abnormalities in COPD group; the latter were correlated with patient characteristics and Mini–Mental Status Examination Questionnaire (MMSEQ) scores to seek any significant correlation.

**RESULTS:**

Twenty-six (65%) of the 40 COPD patients had BAEP abnormalities. We observed significantly prolonged latencies of waves I, III, V over left ear and waves III, IV, V over right ear; increased IPLs of I-V, III-V over left ear and of I-III, I-V, III-V over right side. Amplitudes of waves I-Ia and V-Va were decreased bilaterally. Over left ear, the latencies of wave I and III were significantly correlated with FEV_1_; and amplitude of wave I-Ia, with smoking pack years. A weak positive correlation between amplitude of wave I-Ia and duration of illness; and a weak negative correlation between amplitude of wave V-Va and MMSEQ scores were seen over right side.

**CONCLUSIONS:**

We observed significant subclinical BAEP abnormalities on electrophysiological evaluation in studied stable COPD male patients having mild-to-moderate airflow obstruction.

Chronic obstructive pulmonary disease (COPD) is a disease state characterized by airflow limitation that is not fully reversible. The airflow limitation is usually both progressive and associated with an abnormal inflammatory response of the lungs to noxious particles or gases. COPD is a major public health problem and, currently, fourth leading cause of death worldwide.[1] A further increase in prevalence of, and mortality due to, the disease is predicted for the coming decades. COPD is presently regarded as a multi-system disorder. The associated peripheral neuropathy is well described in medical literature.[2,3] In addition, motor neuron involvement, encephalopathy, and derangement of cognitive function have been observed in patients with chronic respiratory insufficiency. Brainstem auditory evoked potentials (BAEP) are the potentials recorded from the ear and vertex in response to a brief auditory stimulation to assess the conduction through auditory pathway up to midbrain. BAEP in patients with COPD have been evaluated in previous studies, but the characteristics of included patients and study outcomes have been at great variation.[4–6] Kayacan *et al.* observed that smoking, airways obstruction, and long-lasting COPD may not only cause peripheral neuropathy but may also affect the ponto-medullary portion of the brain due to hypoxemia, hypercapnia, and respiratory acidosis.[4] Atis and co-workers studied BAEP in patients with severe COPD and concluded that eighth cranial nerve and brainstem functions were impaired in COPD.[5] Barbieri *et al.* reported that there was no significant difference in BAEP in mild-or-moderate chronic respiratory insufficiency, apart from acidosis.[6] It appears the previous studies have included COPD patients having severe airflow obstruction or significant hypoxemia/hypercapnia. The present study is undertaken to find out prevalence of BAEP abnormalities in stable patients with COPD having no clinical auditory dysfunction/impairment; and to analyze for possible correlation of BAEP abnormalities with patient characteristics, including age, duration of illness, quantum of smoking, spirometric indices, and Mini–Mental Status Examination Questionnaire scores.

## Materials and Methods

The study was conducted in the departments of Respiratory Medicine and Physiology at Rohtak, India. This was a cross-sectional study and was approved by the Institutional Board of Studies and by the ethical committee. All subjects were male and enrolled between November 2006 and October 2007. The COPD patients fulfilling the criteria of the study, having age at least 40 years, attending the COPD clinic run at the Department of Respiratory Medicine, and who gave consent to complete the required investigations as per study protocol were included in the study. The diagnosis of COPD was based on the modified criteria defined in the Global Initiative for Chronic Obstructive Lung Disease (GOLD) guidelines.[7] All the included COPD patients were smokers and had irreversible/partially reversible obstruction of airflow. The patients were included only if they had a stable course of their disease with regular follow-up during the preceding 1 year and no hospitalization for COPD-related illness during the preceding 6 months. Patients with *clinical evidence* of any neurological deficit/neuropathy or those having concomitant diabetes mellitus, chronic alcoholism, uremia, cystic fibrosis, sarcoidosis, leprosy, malignancy, any hereditary disorders involving peripheral nerves, history of intake of any neurotoxic drug, or history of any traumatic lesion possibly affecting brainstem functions were excluded from the study. The control group comprised of an equal number of age-matched healthy volunteers having no risk factor that may lead to neuropathy. All healthy volunteers were nonsmokers. They were selected from medical/paramedical staff of our institute; some healthy attendants of the patients were also included in the control group.

Smoking pack years were calculated from mode of smoking (*bidi,* cigarette, or *hookah*), daily consumption, and the total number of years for which the patient had been smoking. One pack year was 20 cigarettes smoked everyday for 1 year.[8] For *bidi,* cigarette equivalents were calculated by applying a factor of 0.5 to the number of *bidis*;[9] and for hookah, 12.5 g of loose tobacco was equivalent to one packet of 20 cigarettes.[10]

The spirometry was carried out on Transfer Test Model ‘C’ (P. K. Morgan, Kent, UK). Inhaled short-acting bronchodilators were withheld for 6 hours before the test; long-acting β-agonists, 12 hours before the test; and sustained-release theophyline, 24 hours before the test. Spirometric indices were calculated using the best out of 3 technically satisfactory performances as per recommendations of the American Thoracic Society.[11] The following parameters were recorded: peak expiratory flow rate (PEFR), forced expiratory volume in the first second (FEV_1_), forced vital capacity (FVC), and FEV_1_/FVC%.

Electrophysiological studies were carried out on a computerized nerve conduction testing equipment: RMS EMG EP MARK II (Recorders and Medicare Systems Pvt. Ltd., Chandigarh, India); the settings were as shown in Table 1.

**Table 1:** Various settings for brainstem auditory evoked potentials

Stimulus parameter:
Click stimuli having intensity 70 dB above normal hearing threshold was presented to both ears monaurally. During stimulation of one ear, the other ear was masked by 40-dB sound. A total of 2000 stimulations generated by passing 0.1-millisecond square pulses through shielded headphones with alternating polarity were applied on both ears. Stimuli were at the rate of 11.1/s.
Filters:
1. Low: 100 Hz
2. High: 3 KHz
Stimulus polarity:
Two types of clicks were produced: one, moving the earphone diaphragm away from eardrum (rarefaction click); and the other, moving it in the opposite direction (condensation or compression click). In this study, stimulus with alternating polarity was used.
Recording electrodes:
The volume-conducted evoked responses are picked up from scalp by electrodes. Two reference electrodes were attached to left and right mastoids, designated as A1 and A2 respectively; one active electrode on vertex, labeled as Cz; and one as ground electrode to forehead, termed as Fz. All the electrodes were plugged to the junction box. Skin-to-electrode impedance was monitored and kept below 5 KΩ.
Recommended montage for BAEP:
Channel I: Cz-A1
Channel 2: Cz-A2
Ground: Fz

### Procedure of BAEP

The patient was put at ease and was made to lie down with eyes closed, relaxed on a couch, in a soundproof and air-conditioned room. After thorough cleaning of the electrode recording sites on the scalp, electrolyte paste was applied on the recording surface of disk electrodes and then Ag/AgCl electrodes were affixed at predetermined positions on the scalp according to 10/20 international system of electrode placement.[12] The signals were picked by electrodes and were filtered, amplified, averaged, displayed on the screen of RMS EMG EP MK2, and recorded. Subsequently, interpeak latencies (IPLs) were calculated.

**Figure 1:**
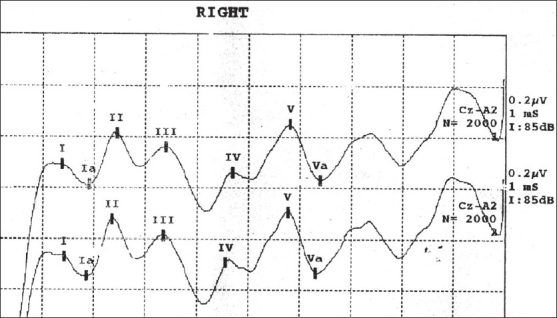
Brainstem auditory evoked potentials wave pattern over right ear of a healthy volunteer. Wave I and IPL of I-III represent the peripheral part of the pathway; whereas wave III and IPL of III-V, the central part

The normal BAEP recording consists of five or more vertex-positive and vertex-negative waves [Figure 1] arising within 10 milliseconds of auditory stimulus.[13] Latencies of waves I, II, III, IV, and V, together with interpeak latencies of I-III, I-V, and III-V, and amplitudes of waves I and V were measured from recordings.

### Mini-mental status examination

All included subjects, including COPD patients and healthy volunteers, were analyzed for their mental status using the Mini–Mental Status Examination Questionnaire (MMSEQ).[14]

### Statistical analyses

The data of healthy volunteers and COPD patients was analyzed by incorporating the same in two different groups. The data were examined for normal distribution, and transformations were made where appropriate. The group means and the standard deviations for each variable were calculated in healthy volunteers group and COPD group separately. The statistical significance of difference between group means of various parameters between healthy volunteers group and COPD group was analyzed by using independent sample *t* test, and a *P*value <.05 was considered statistically significant. Individual COPD patients having BAEP abnormality beyond the range of ‘mean ± 3’ standard deviation from healthy volunteers were considered as having significant BAEP abnormality. The BAEP abnormalities in COPD patients were correlated with patients' characteristics, including age, duration of illness, quantum of smoking, spirometric indices (FEV_1_, FEV_1_/FVC%, and PEFR), and the MMSEQ scores. The data obtained was statistically analyzed using Pearson's correlation. All statistical analyses were carried out with the help of SPSS (version 14.0), Chicago, software.

## Results

We included 80 male subjects comprising of 40 COPD patients and 40 age-matched healthy volunteers. All subjects were aged 40 years or more. The characteristics of subjects included in the present study were as shown in Table 2. The COPD patients had a post-bronchodilator FEV_1_ less than 80% of the predicted value, along with an FEV_1_/FVC% not more than 70%. They had an increase in FEV_1_ less than 200 mL, or less than 12% of baseline value 20 minutes after 2 puffs of inhaled salbutamol given via a metered-dose inhaler using a spacer. The duration of symptoms in all patients with COPD was 5 years or more. All healthy volunteers were nonsmokers and had no symptoms suggestive of any disease. As expected, their spirometric indices were statistically different from COPD patients.

**Table 2:** Characteristics of subjects in COPD group [*n* = 40] and healthy volunteers group [*n* = 40]

	COPD group mean±SD	Healthy volunteers group mean±SD	*P*-value
Age	57.25±9.07	56.9±9.21	0.09
Duration of illness (yrs)#	10.67±4.89	Nil	-
Smoking (Pack years)#	39.95±20.94	Nil	-
Height(m)	1.677±.004	1.66±.005	0.142
PEFR(L)	3.42±1.27	7.59±0.30	<0.001*
FEV_1_(I/min)	1.48±0.50	2.90±0.12	<0.001*
FVC(I/min)	2.77±0.66	3.48±0.14	<0.001*

* *P* < .05 — significant result.

# As a prerequisite in our study protocol, healthy volunteers were asymptomatic and nonsmokers.

Table 3 provides summaries [mean ± SD] of variables of BAEP wave patterns recorded over left ear and right ear separately in healthy volunteers group, comparing the same with those in COPD group. Over left ear, the latencies of waves I, III, and V in COPD patients were prolonged significantly as compared to the healthy volunteers. The latencies of waves II and IV were also increased in COPD group but had no statistical significance. Over the right side, there was significant prolongation of the latencies of waves III, IV, and V in COPD group as compared to the healthy volunteers.

**Table 3:** Brainstem auditory evoked potentials variables in COPD patients [*n* = 40] and healthy volunteers [*n* = 40]

BAEP variables (unit)	Left ear			Right ear		
		
	COPD group mean±SD	Healthy volunteers group mean±SD	*P*-value	COPD group mean±SD	Healthy volunteers group mean±SD	*P*-value
Latencies						
I (ms)	1.72±0.30	1.57±0.25	<0.001*	1.43±0.18	1.41±0.10	0.57
II (ms)	2.59±0.21	2.54±0.30	0.30	2.81±0.21	2.47±0.21	0.88
III (ms)	3.79±0.37	3.58±0.18	<0.001*	3.73±0.38	3.36±0.14	<0.001*
IV (ms)	4.59±0.25	4.58±0.27	0.79	4.47±0.35	4.38±0.26	<0.001*
V (ms)	5.91±0.40	5.29±0.39	0.001*	5.85±0.36	5.27±0.21	<0.001*
Interpeak latencies						
I-III (ms)	2.06±0.32	2.00±0.17	0.30	2.30±0.38	1.96±0.16	<0.001*
I-V (ms)	4.13±0.55	3.72±0.36	<0.001*	4.41±0.34	3.85±0.23	<0.001*
III-V (ms)	1.72±0.31	2.11±0.41	<0.001*	2.11±0.34	1.91±0.15	<0.001*
Amplitudes						
I-Ia (μv)	0.32±0.26	0.66±0.71	<0.001*	0.27±0.34	0.29±0.13	<0.001*
V-Va (μv)	0.45±0.57	0.49±0.13	<0.001*	0.39±0.39	0.42±0.16	<0.001*

* The difference between the two groups was statistically significant.

The interpeak latencies (IPLs) of III-V and I-V were significantly prolonged in the COPD patients as compared to healthy volunteers over both ears; in addition, interpeak latency of I-III was significantly prolonged in the COPD group over right ear.

Amplitude of the wave I-Ia in the COPD patients was significantly decreased when compared to that in healthy volunteers, over both ears respectively. Similarly, amplitude of the wave V-Va in the COPD patients was significantly decreased when compared to that in healthy volunteers, over both ears respectively.

Individual COPD patients who had any BAEP abnormality were also analyzed, and the details are shown in Table 4. The BAEP abnormality was considered to exist when there was prolongation of any latency or interpeak latency beyond 3 times the standard deviation of healthy volunteers and/or a decrease in any amplitude beyond 3 times the standard deviation of healthy volunteers [99^th^ percentile]. Prolongation of latency of wave III was most common; followed by prolongation of IPL of I-V, latency of wave V, IPL of I-III, and IPL of III-V. In total, 26/40 [65%] COPD patients had abnormalities in one or more BAEP variables; 24/40 [60%] patients had BAEP abnormalities in the form of increased latency of waves I, II, III, IV, and V, and an equal number of patients had BAEP abnormalities in the form of increased IPL of I-III, I-V, and III-V. The decrease in amplitude for wave V-Va was noted in 7/40 [17.5%] patients; and that for wave I-Ia, in 5/40 [12.5%] patients.

**Table 4:** Individual COPD patients having BAEP abnormalities (abnormality defined as a value beyond ±3 SD from mean for the healthy volunteer group)

		Patients with BAEP abnormalities over left ear		Patients with BAEP abnormalities over right ear		Patients with BAEP abnormalities any one or both side	
				
		n	Percentage	n	Percentage	n	Percentage
Latencies	I	2	5	1	2.5	3	7.5
	II	0	-	1	2.5	1	2.5
	III	6	15	19	47.5	20	50
	IV	0	-	1	2.5	1	2.5
	V	4	10	14	35	15	37.5
Interpeak latencies	IPL(I-III)	2	5	14	35	14	35
	IPL(I-V)	5	12.5	16	40	18	45
	IPL(III-V)	1	2.5	7	17.5	8	20
Amplitude	Amp I-Ia	1	2.5	1	2.5	2	5
	Amp V-Va	5	12.5	3	7.5	7	17.5

**Figure 2:**
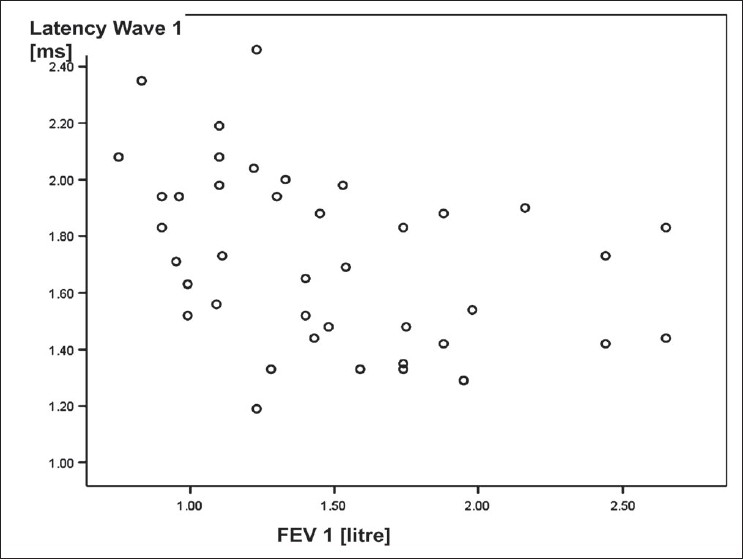
Scattered plot diagram showing a negative correlation between FEV1 and latency of wave 1 of BAEP wave pattern recorded over left ear in COPD patients

**Figure 3:**
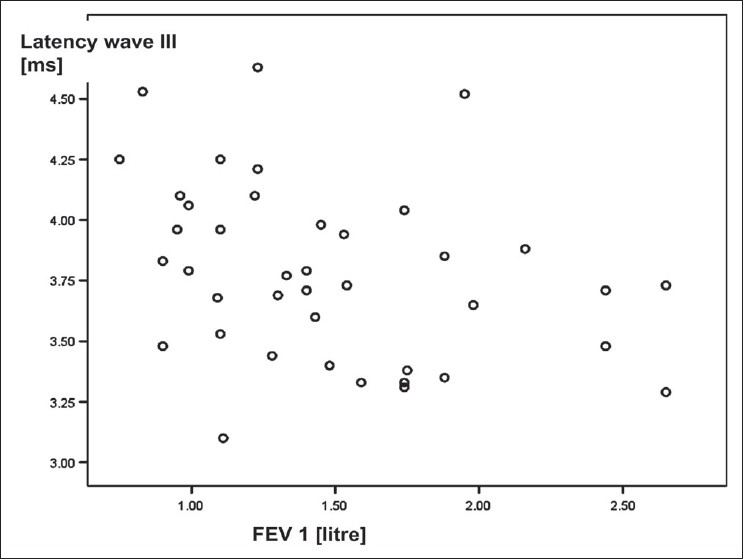
Scattered plot diagram illustrating a negative correlation between FEV1 and latency of wave III of BAEP wave pattern recorded over left ear in COPD patients

**Figure 4:**
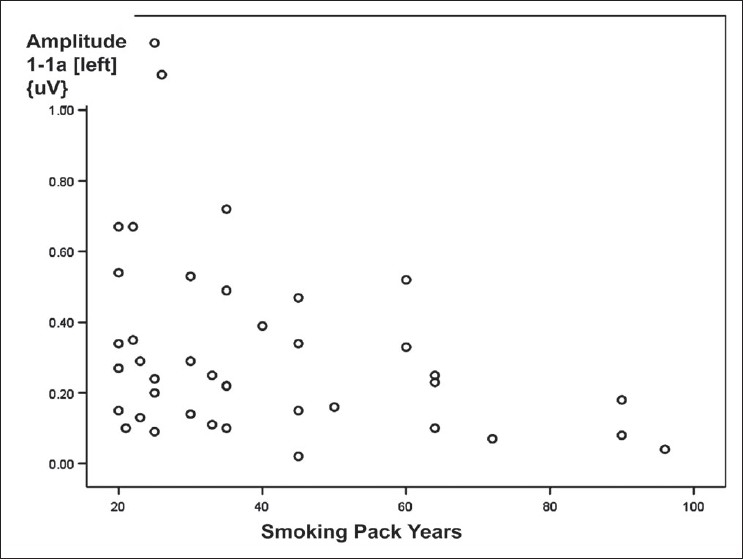
Scattered plot diagram showing a weak negative correlation was observed between smoking pack years and amplitude of wave I-Ia of BAEP wave pattern recorded over left ear in COPD patients

Table 5 shows correlation between BAEP variables observed over *left side* and the characteristics of COPD patients. The latencies of wave I of BAEP wave pattern recorded over left ear correlated negatively with FEV_1_; the correlation was statistically significant [Figure 2]. Similarly, the latencies of wave III over left ear correlated negatively with FEV_1_; the correlation was statistically significant [Figure 3]. The correlation between amplitude of wave I-Ia recorded over left ear and smoking pack years was a negative one and statistically significant [Figure 4]. Other correlations were not statistically significant.

**Table 5:** Correlation of variables of BAEP wave patterns recorded over left ear with age, duration of illness, pack years, spirometric indices, and MMSEQ scores

BAEP (Lt)		Age	Duration of illness	Pack years	PEFR	FEV_1_	FEV_1_/FVC	MMSEQ score
I (ms)	r	0.114	0.045	0.042	-0.228	-0.377	-0.221	0.031
	p	0.485	0.781	0.797	0.156	0.001*	0.172	0.851
III (ms)	r	0.072	0.097	0.137	-0.198	-0.331	-0.247	-0.015
	p	0.658	0.550	0.401	0.220	0.001*	0.125	0.926
V (ms)	r	0.085	0.054	0.125	-0.252	-0.234	-0.181	-0.260
	p	0.601	0.739	0.443	0.117	0.147	0.263	0.105
I-III (ms)	r	0.028	0.071	0.116	-0.006	-0.016	-0.070	-0.046
	p	0.866	0.665	0.477	0.971	0.924	0.668	0.780
I-V (ms)	r	0.097	0.058	0.121	-0.208	-0.024	-0.076	-0.327
	p	0.551	0.721	0.456	0.198	0.883	0.641	0.039
III-V (ms)	r	0.004	0.031	0.180	-0.055	0.097	0.018	-0.241
	p	0.982	0.851	0.266	0.736	0.552	0.911	0.135
Amp I-Ia	r	0.274	0.263	-0.340	0.292	0.173	0.180	0.001
	p	0.087	0.102	0.032*	0.068	0.285	0.267	0.995
Amp V-Va	r	0.235	0.136	-0.108	-0.157	-0.069	-0.081	-0.218
	p	0.144	0.402	0.508	0.332	0.672	0.619	0.177

* Correlation is significant at the 0.05 level (2-tailed). r = Pearson's coefficient. p = *P* value.

**Figure 5:**
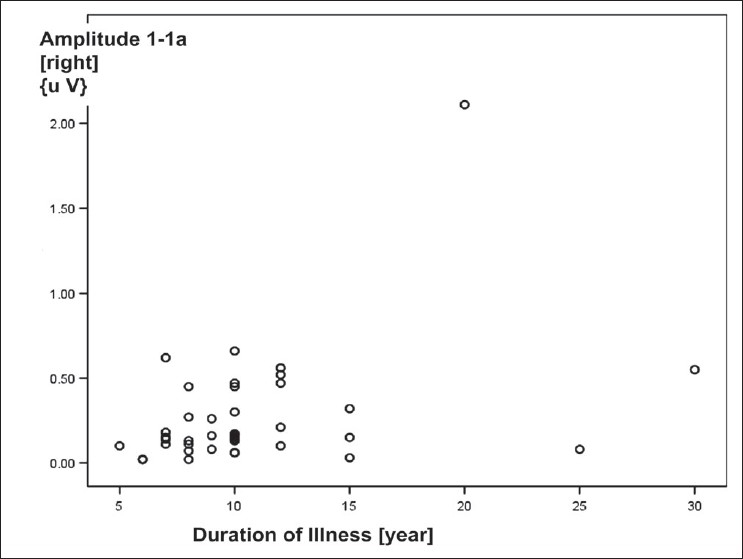
Scattered plot diagram showing a positive correlation was seen between duration of illness [due to COPD] and amplitude of wave I-Ia of BAEP wave pattern recorded over right ear in COPD patients

**Figure 6:**
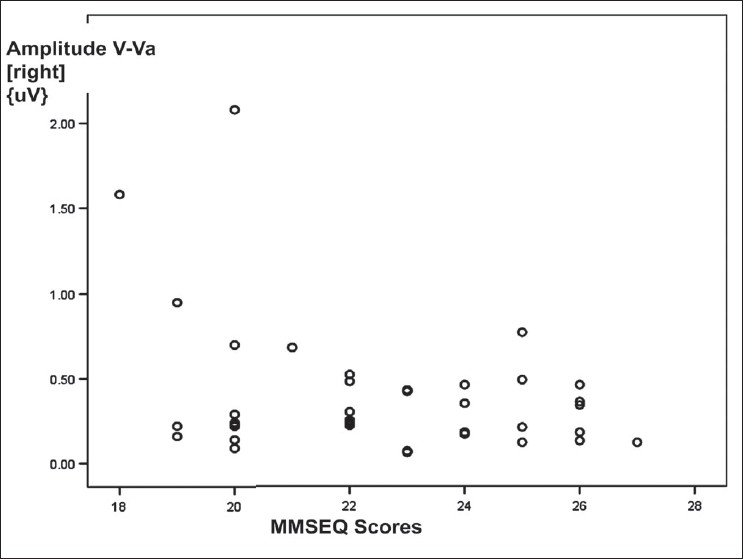
Scattered plot diagram showing a weak negative correlation between Mini–Mental Status Examination Questionnaire scores and amplitude of wave V-Va of BAEP wave pattern recorded over right ear in COPD patients

The correlations between the variables of BAEP wave patterns recorded over right ear and the characteristics of COPD patients were as shown in Table 6. The correlation between amplitude of wave I-Ia and duration of illness was a weak positive one [Figure 5]; the correlation between amplitude of wave V-Va and MMSEQ scores was a weak negative one [Figure 6]; though both were statistically significant. Other correlations between BAEP variables and the characteristics of COPD patients were not significant.

**Table 6:** Correlation of variables of BAEP wave patterns recorded over right ear with age, duration of illness, pack years, spirometric indices, and MMSEQ scores

BAEP (Rt)		Age	Duration of illness	Pack years	PEFR	FEV_1_	FEV_1_/FVC	MMSEQ score
I	r	0.173	0.308	0.263	-0.211	-0.242	-0.211	0.154
	p	0.285	0.053	0.101	0.191	0.133	0.192	0.342
II	r	0.58	0.221	0.101	-0.197	-0.276	-0.063	-0.127
	p	0.722	0.170	0.535	0.223	0.084	0.698	0.436
III	r	0.044	0.071	0.050	-0.211	-0.206	-0.044	-0.165
	p	0.787	0.662	0.759	0.190	0.203	0.787	0.310
IV	r	0.024	0.010	-0.143	-0.094	-0.186	-0.121	-0.223
	p	0.894	0.952	0.377	0.563	0.251	0.456	0.167
V	r	0.051	0.183	0.019	-0.094	-0.219	-0.145	-0.009
	p	0.757	0.259	0.907	0.591	0.171	0.406	0.995
I-III	r	0.022	0.086	0.153	-0.201	-0.213	-0.061	-0.179
	p	0.895	0.596	0.346	0.214	0.187	0.709	0.270
I-V	r	0.107	0.027	0.118	-0.021	-0.142	-0.068	-0.099
	p	0.513	0.870	0.469	0.896	0.382	0.676	0.544
III-V	r	0.103	0.113	0.029	0.131	0.004	0.104	0.185
	p	0.529	0.489	0.860	0.419	0.982	-0.521	0.254
Amp I-Ia	r	0.109	0.380	-0.262	-0.154	-0.151	-0.031	0.042
	p	0.502	0.016*	0.103	0.342	0.353	0.850	0.796
Amp V-Va	r	0.194	0.012	0.185	-0.201	-0.039	0.097	-0.319
	p	0.230	0.941	0.252	0.213	0.813	0.554	0.045*

* Correlation is significant at the 0.05 level (2-tailed). r = Pearson's coefficient. p = *P value*.

## Discussion

Before we discuss and compare the observations in our study with those of other studies, we feel it is worthwhile to consider significant differences between characteristics of the study subjects included in our study and those of the subjects in other studies [Table 7]. Kayacan *et al.*[4] included 32 patients with COPD having age 61 ± 8.8 years. They have not described the details of the inclusion and irreversibility criteria. Atis *et al.*[5] included 21 patients with severe COPD according to the criteria[15] of the American Thoracic Society (1987). Some of the patients included had clinical evidence of neuropathy. In our study, all COPD patients were significant smokers and had irreversible/partially reversible airflow limitation, a defining characteristic of COPD. Other studies did not have conformity regarding the reversibility criteria as recommended in Global Initiative for Chronic Obstructive Lung Disease (GOLD) guidelines,[7] which were taken into consideration in the present study. Moreover, quantum of smoking in our study was more despite a lower mean age of COPD patients when compared to that in previous two studies.

**Table 7:** Comparison between previous studies and our study

Study	No. of study subjects	COPD patients characteristics	BAEP parameters studied	Patients with BAEP abnormalities	BAEP parameters affected	Correlations
Kayacan *et al.*[4]	32 COPD subjects [male=30];	19/32 had PaO_2_<55mmHg	Latencies I, II, III, IV, V	Individual patients data not mentioned	Latency III	III ** PEFR, FEF25,
	no controls	Age=61±8.8 years;	IPL I-III, III-V, I-V		IPL I-III, III-V	FEF25-75
		smoking pack				IV** FVC, PEFR
		years=37.4±28.5				IPL I-III**
						FEF25-75, FEV_1_/FVC,
						FEF25-75
						IPL III-V** PaCO_2_,
						HCO_3_, pH
Atis *et al.*[5]	21 COPD patients [male=16]	Severe airflow obstruction;	Latencies I, III, V	16/21 [76.1%]	Latency I, V	No significant
	11 had clinical neuropathy	Age=64±6.5 years;	IPL I-III, III-V, I-V		IPL III-V, I-V	Correlation with pH, PaO_2_,
	controls=21	Only 15/21 smokers,	Amplitude wave I, III, V			PaCO_2_, FEV_1_%
		pack years = 24.59±21.21;				FEV_1_/FVC,
		FEV_1_=0.96+0.32				duration of disease or cigarette consumption
Our study	COPD patients =40, all male all male	Stable COPD patients,	Latencies I, II, III, IV, V	26/40 [65%]	Latency I, III, IV, V	Left side:
	[None had clinical neurological deficiency]	Age = 57.25±9.07	IPL I-III, III-V, I-V		IPL I-III, III-V, I-V	I, III** FEV_1_
	Healthy volunteers=40,	Smoking pack	Amplitude I-Ia, V-Va		Amplitude I-Ia, V-Va	Amplitude I-Ia** smoking
	all male	years= 39.95±20.94				Right side:
		FEV_1_= 1.48±0.50				Amplitude I-Ia**
						Duration of illness

Significant correlations between variables are shown by [**].

In our study, we included stable COPD patients with mild-to-moderate airflow obstruction and with no clinical features suggestive of any neuropathy. Our objective was to assess the impaired brainstem auditory evoked potentials in *stable* COPD patients [and perhaps early in the course of their disease] with *no clinical features of any neurological deficiency* — the COPD patients that are usually seen at the level of general clinical practice. This study group was not evaluated in previous studies. It is not reasonable to compare prevalence of peripheral neuropathy observed in our study with that observed in other previous studies due to differences in the characteristics of subjects included in various studies.

The data analysis of individual COPD patients in our study showed that, overall, 26/40 [65%] COPD patients had abnormalities in one or more BAEP variables; 24/40 [60%] patients had BAEP abnormalities in the form of increased latency of waves I, II, III, IV, and V; and an equal number of patients had BAEP abnormalities in the form of increased interpeak latencies of I-III, I-V, and III-V. The decrease in amplitude of wave V-Va was noted in 7/40 [17.5%] patients; and that in amplitude of wave I-Ia, in 5/40 [12.5%] patients. All previous studies have suggested the existence of BAEP abnormalities in patients with COPD, though prevalence varied from one study to the other study [Table 7], the possible explanation being nonuniformity between study subjects from different studies. The studies that have included some patients having clinical evidence of brain stem involvement have reported higher prevalence of BAEP abnormalities on neurophysiologic investigations. Similarly, patients with severe hypoxemia and/or hypercapnia had a higher prevalence of BAEP abnormalities. Atis *et al.* noted BAEP abnormalities in 76.1% of COPD patients. Our study found 65% of COPD patients with abnormal BAEP values.

The common BAEP abnormalities observed in COPD patients in our study and previous studies include prolongation of latencies of waves I, III, V; and interpeak latencies of I-III and III-V. In addition, our study found decreased amplitudes of waves I-Ia and V-Va. Though none of the COPD patients included in the present study had significant hypoxemia or hypercarbia, the existing medical literature suggests that hypoxemia results in peripheral nerve damage by harming the vaso nervosum. In the early stages of ischemia, mechanisms to reduce peripheral neuropathy are activated, but these become insufficient over time and obvious neuropathy is inevitable in chronic hypoxemia.[16] It has been hypothesized that the abnormal BAEP findings are due to brainstem hypoxia which increases with the severity of COPD. Sohmer *et al.* demonstrated depression of the auditory nerve–brainstem evoked response, as well as vestibular and visual evoked potentials during severe hypoxemia in cats.[17] In addition to chronic hypoxemia and hypercapnia, other associated factors in patients with COPD, including tobacco smoking; malnutrition; and drugs used in COPD treatment, like long-acting inhaled β2 agonists, inhaled anticholinergic agents, inhaled glucocorticoids, and sustained release theophyline, may be possibly associated with neuropathy seen in COPD patients.[16,18,19] Though none of our patients had significant hypoxemia, they had longer duration of illness and more smoking pack years; so, whether severity of hypoxemia alone or the chronicity and severity of hypoxemia together contribute to development of peripheral neuropathy needs to be evaluated in future studies. As COPD patients in our study were heavy smokers, the possibility of the contents of cigarette smoke leading to BAEP abnormalities remains.

We could not find any correlation between the BAEP parameters and pulmonary function test parameters, except BAEP latency of waves I and III with FEV_1_ on left side. The poor correlation in spite of significant BAEP abnormalities is probably due to the narrow range of patients' characteristics and pulmonary function parameters in our patients as we included relatively stable patients during the early course of COPD, having mild-to-moderate airflow obstruction.

To conclude, in the present study, we observed significant BAEP abnormalities on electrophysiological evaluation in 26/40 [65%] studied stable male COPD patients with mild-to-moderate airflow obstruction (and with no clinical neuropathy), and these patients had significant smoking history with no significant hypoxia or hypercapnia.
